# Supplementary figures and images for: Global Disease Monitoring and Forecasting with Wikipedia
Source: PLoS Comput Biol. 2014 Nov 13;10(11):e1003892. doi: 10.1371/journal.pcbi.1003892 (PMC4231164; doi:10.1371/journal.pcbi.1003892)

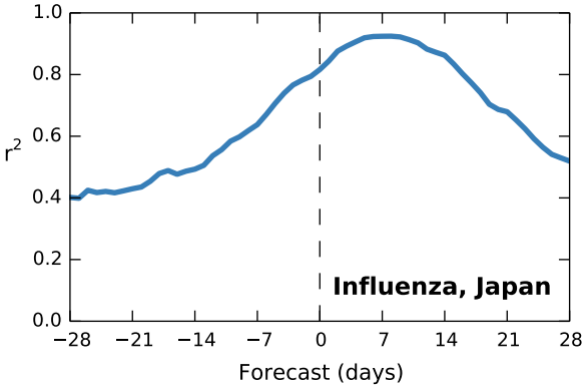

Supplement: Dataset S1 — Input data, raw results, and additional figures. This archive file contains: (a) inter-language article mappings, (b) figures for the 4 successful contexts not included above, (c) official epidemiological data used as input, (d) complete correlation scores r, (e) wiki input data, and (f) a text file explaining the archive content and file formats. (ZIP) [file pcbi.1003892.s001.zip › S1_supplemental-data/figures/lag_ja_flu_2010-06-26_2013-06-29.pdf]

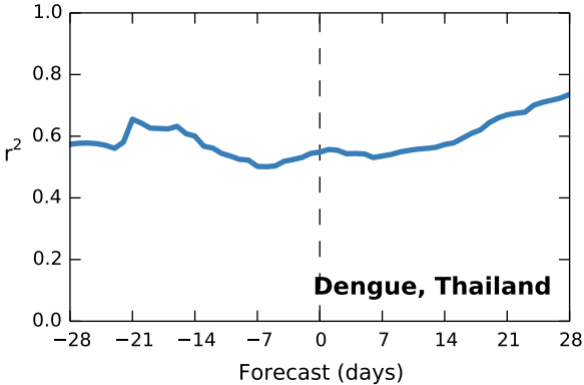

Supplement: Dataset S1 — Input data, raw results, and additional figures. This archive file contains: (a) inter-language article mappings, (b) figures for the 4 successful contexts not included above, (c) official epidemiological data used as input, (d) complete correlation scores r, (e) wiki input data, and (f) a text file explaining the archive content and file formats. (ZIP) [file pcbi.1003892.s001.zip › S1_supplemental-data/figures/lag_th_dengue_2011-01-01_2014-01-01.pdf]

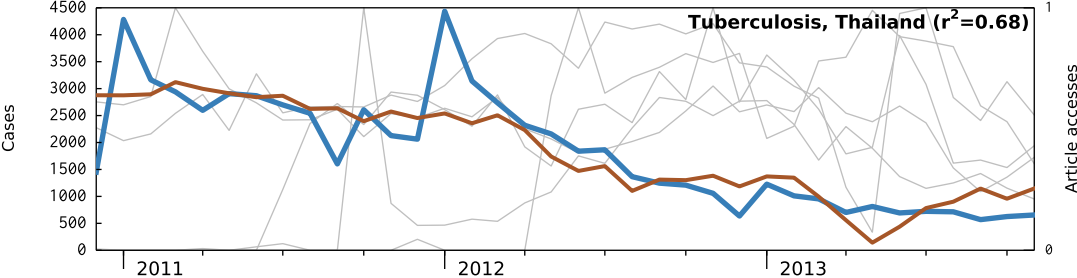

Supplement: Dataset S1 — Input data, raw results, and additional figures. This archive file contains: (a) inter-language article mappings, (b) figures for the 4 successful contexts not included above, (c) official epidemiological data used as input, (d) complete correlation scores r, (e) wiki input data, and (f) a text file explaining the archive content and file formats. (ZIP) [file pcbi.1003892.s001.zip › S1_supplemental-data/figures/incidence_model_accesses_th_tuberculosis_2010-12-01_2013-12-01.pdf]

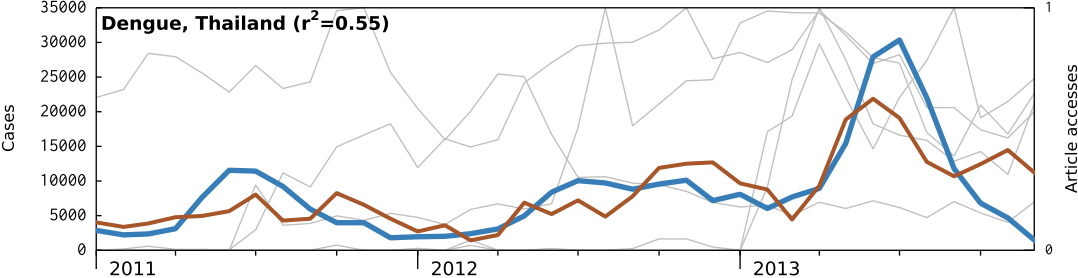

Supplement: Dataset S1 — Input data, raw results, and additional figures. This archive file contains: (a) inter-language article mappings, (b) figures for the 4 successful contexts not included above, (c) official epidemiological data used as input, (d) complete correlation scores r, (e) wiki input data, and (f) a text file explaining the archive content and file formats. (ZIP) [file pcbi.1003892.s001.zip › S1_supplemental-data/figures/incidence_model_accesses_th_dengue_2011-01-01_2014-01-01.pdf]

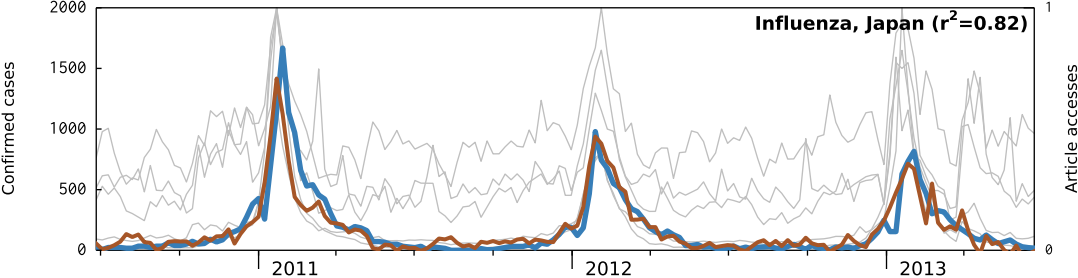

Supplement: Dataset S1 — Input data, raw results, and additional figures. This archive file contains: (a) inter-language article mappings, (b) figures for the 4 successful contexts not included above, (c) official epidemiological data used as input, (d) complete correlation scores r, (e) wiki input data, and (f) a text file explaining the archive content and file formats. (ZIP) [file pcbi.1003892.s001.zip › S1_supplemental-data/figures/incidence_model_accesses_ja_flu_2010-06-26_2013-06-29.pdf]

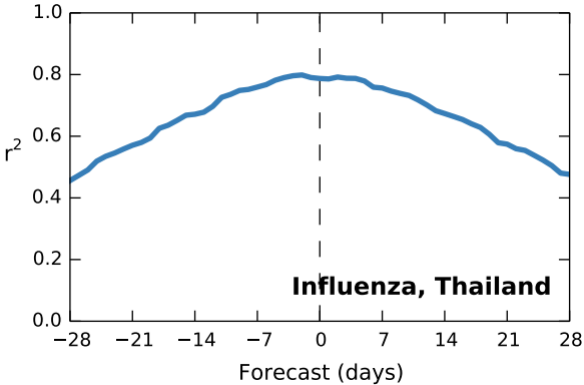

Supplement: Dataset S1 — Input data, raw results, and additional figures. This archive file contains: (a) inter-language article mappings, (b) figures for the 4 successful contexts not included above, (c) official epidemiological data used as input, (d) complete correlation scores r, (e) wiki input data, and (f) a text file explaining the archive content and file formats. (ZIP) [file pcbi.1003892.s001.zip › S1_supplemental-data/figures/lag_th_flu_2011-01-23_2014-01-26.pdf]

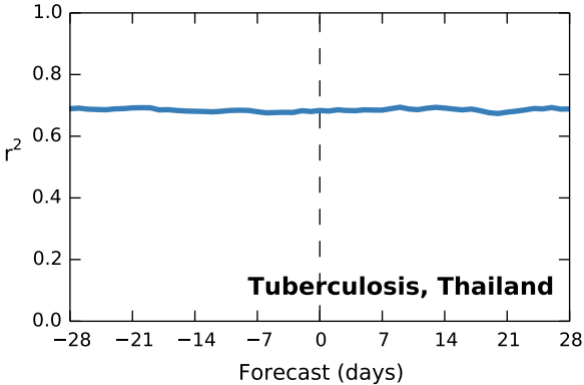

Supplement: Dataset S1 — Input data, raw results, and additional figures. This archive file contains: (a) inter-language article mappings, (b) figures for the 4 successful contexts not included above, (c) official epidemiological data used as input, (d) complete correlation scores r, (e) wiki input data, and (f) a text file explaining the archive content and file formats. (ZIP) [file pcbi.1003892.s001.zip › S1_supplemental-data/figures/lag_th_tuberculosis_2010-12-01_2013-12-01.pdf]

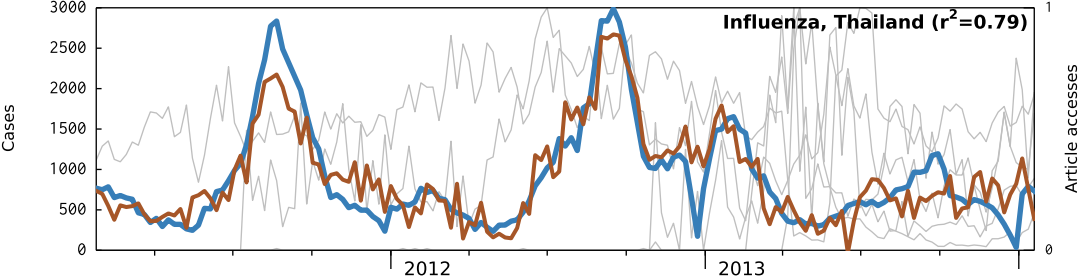

Supplement: Dataset S1 — Input data, raw results, and additional figures. This archive file contains: (a) inter-language article mappings, (b) figures for the 4 successful contexts not included above, (c) official epidemiological data used as input, (d) complete correlation scores r, (e) wiki input data, and (f) a text file explaining the archive content and file formats. (ZIP) [file pcbi.1003892.s001.zip › S1_supplemental-data/figures/incidence_model_accesses_th_flu_2011-01-23_2014-01-26.pdf]
